# Medical follow-up of workers exposed to lung carcinogens: French evidence-based and pragmatic recommendations

**DOI:** 10.1186/s12889-017-4114-1

**Published:** 2017-02-14

**Authors:** Fleur Delva, Jacques Margery, François Laurent, Karine Petitprez, Jean-Claude Pairon, RecoCancerProf Working Group, Michel André, Dominique Bessette, Patrick Brochard, Jean-François Certin, Christos Chouaid, Bénédicte Clin-Godard, Pierre Goutet, Philippe Grenier, Gladys Ibanez, Yuriko Iwatsubo, Claudie Lebaupain, Chloë Leroy, Bernard Milleron, Christophe Paris, Isabelle Stücker, Gilbert Thouveny, Dominique Tirmarche, Martine Vandame, Odile Vandenberghe

**Affiliations:** 1 Univ. Bordeaux, Inserm, Bordeaux Population Health Research Center, team HEALTHY, UMR 1219, Bordeaux, F-33000 France; 2 CHU de Bordeaux, Pole de sante publique, Service de médecine du travail et de pathologies professionnelle, F-33000 Bordeaux, France; 3 Clinical epidemiology and research, Institute Bergonié, Bordeaux, France; 4 Respiratory Medicine Department, Percy Military Hospital, Clamart, France; 5 French Military Health Service Academy, École du Val de Grâce, Paris, France; 6 Groupe d’Oncologie de Langue Française (GOLF), Société de Pneumologie de Langue Française (SPLF), Paris, France; 7 Department of Cardiovascular Imaging, Hôpital Cardiologique du Haut-Lévêque, CHU de Bordeaux, Pessac, France; 8 Institut Liryc/Equipex Music, Université de Bordeaux-Inserm U1045, Pessac, France; 9 Société de Radiologie Française (SFR), Paris, France; 10 Service des bonnes pratiques professionnelles, Haute Autorité de Santé (HAS), Saint Denis-La Plaine, France; 11 INSERM U955, Université Paris Est Créteil, Créteil, France; 12 Institut Santé-Travail Paris-Est, Centre Hospitalier Intercommunal, Créteil, France; 13 Société Française de Médecine du Travail (SFMT), Paris, France

**Keywords:** Lung neoplasms, Cancer screening, Recommendations

## Abstract

**Background:**

The aim of this work was to establish recommendations for the medical follow-up of workers currently or previously exposed to lung carcinogens.

**Methods:**

A critical synthesis of the literature was conducted. Occupational lung carcinogenic substances were listed and classified according to their level of lung cancer risk. A targeted screening protocol was defined.

**Results:**

A clinical trial, National Lung Screnning Trial (NLST), showed the efficacy of chest CAT scan (CT) screening for populations of smokers aged 55–74 years with over 30 pack-years of exposure who had stopped smoking for less than 15 years. To propose screening in accordance with NLST criteria, and to account for occupational risk factors, screening among smokers and former smokers needs to consider the types of occupational exposure for which the risk level is at least equivalent to the risk of the subjects included in the NLST. The working group proposes an algorithm that estimates the relative risk of each occupational lung carcinogen, taking into account exposure to tobacco, based on available data from the literature.

**Conclusion:**

Given the lack of data on bronchopulmonary cancer (BPC) screening in occupationally exposed workers, the working group proposed implementing a screening experiment for bronchopulmonary cancer in subjects occupationally exposed or having been occupationally exposed to lung carcinogens who are confirmed as having high risk factors for BPC. A specific algorithm is proposed to determine the level of risk of BPC, taking into account the different occupational lung carcinogens and tobacco smoking at the individual level.

**Electronic supplementary material:**

The online version of this article (doi:10.1186/s12889-017-4114-1) contains supplementary material, which is available to authorized users.

## Background

In 2012, bronchopulmonary cancer (BPC) was the most frequently observed cancer, with 1.8 million new cases across the globe. It is also the leading cause of death by cancer, with approximately one death out of five, of all cancers combined [1].

In addition to tobacco consumption, occupational exposure to carcinogenic products is another major risk factor of BPC, and review studies estimate that the proportion of BPCs attributable to occupational exposure varies from 13 to 29% in men, the most frequently involved carcinogenic agent being asbestos [2, 3]. Several professional etiologies have been identified for BPC and have been the subject of reviews of the literature [4–6]. The confirmed (IARC group 1) carcinogenic agents (and exposure situations) for which there is over-incidence of BPC include the following^1^: asbestos, arsenic (and arsenic-based compounds), benzo(a)pyrene, beryllium (and beryllium-based compounds), bis(chloromethyl) ether and chloromethyl methyl ether, cadmium (and cadmium-based compounds), hexavalent chromium derivatives, diesel engine emissions, sulfur mustard, coal tar, coal tar pitch, soot, coal gasification and coke production, work in iron and steel foundries, certain nickel derivatives, plutonium-239, radon-222, X-rays and gamma rays and daughter products (work in iron ore mines), crystalline silica, the painting profession, passive smoking, talc containing asbestiform fibers, aluminum production using the Söderberg process, and the rubber industry.

In 70% of cases, BPC presents with immediate metastases, preferentially located in the liver, bone, brain, suprarenal glands and the skin. This frequent metastatic dissemination has an impact on the therapeutic strategy and prognosis. The median survival in patients presenting with clinical stage IA cancer is 58 months and is reduced to 6 months for a patient with stage IV cancer [7].

In France, work regulations provide for the implementation of multidisciplinary occupational health teams working towards the prevention of occupational risks (study of exposure, provision of advice, promotion of occupational health, etc.) and, in cases in which occupational risk is not totally controlled, reinforced medical surveillance for subjects who are occupationally exposed to carcinogenic agents; however, precise details on the modalities of this surveillance are rarely provided. After retirement, the implementation of post-occupational surveillance is also scheduled. It is worth noting that, for many lung carcinogens and for the exposure to such agents, post-occupational surveillance (implemented in 1995 in France) continues to provide for lung X-rays once every 2 years but makes no reference to other imaging techniques (chest CAT (CT) scans in particular). It would therefore appear necessary to reassess the pertinence and frequency of the associated medical examinations involved in the surveillance of subjects exposed to these lung carcinogens. For past asbestos exposure, previous recommendations in 1999 and 2010 have been proposed in France to monitor only benign pleuropulmonary diseases [8, 9]. During post-occupational surveillance, chest CT scans are recommended for asbestos-exposed subjects at a frequency of every 5 to 10 years, depending on the cumulative asbestos exposure after a latency period. In Italy, Mastrangelo et al. proposed in 2013 methods to follow-up workers with past occupational exposure to asbestos [10]. According to results published by the National Lung Screening trial (NLST) (United States) [11], the efficacy of screening by chest CT scan is based on an annual renewal of the examination for populations of smokers with a consumption of over 30 pack-years who have stopped smoking for less than 15 years. Following the publication of the NLST results, recommendations and expert opinions have been published internationally [12–21]. The majority recommends BPC screening by low-dose chest CT scan but in strictly controlled conditions. The pertinence of this type of screening, although assessed in a population of smokers, has not yet been evaluated in populations exposed to other lung carcinogens, in particular occupational carcinogens. The occupational origin of BPC is often difficult to determine within a context of frequently associated tobacco consumption due to the absence of any clinical, histological or evolutive specificity. Although imputability is difficult to establish at the individual level, the identification of occupational exposure to carcinogenic agents is nevertheless important, especially due to the medico-social consequences for the patients, as they can potentially obtain compensation for their condition as an occupational disease. This identification is equally essential for collective prevention in order to reinforce prevention in the workplace in the case of persistent exposure.

The aim of this work is to draft recommendations for the medico-professional surveillance of workers exposed or having been exposed to lung carcinogens using the “Clinical Practice Guidelines” method [22].

## Methods

Subjects included in these recommendations are all workers exposed or having been exposed to lung carcinogens, whether they are active or inactive and regardless of the type of current or former work contract or professional status. The occupational carcinogens studied were occupational carcinogens classified by the WHO (World Health Organization) IARC as carcinogenic to humans (group 1) with sufficient evidence in humans regarding lung cancer.

The subject of our study is vast and raises a number of questions and sub-questions. The available scientific data are highly dispersed and difficult to summarize. In this situation, the most appropriate method, recommended by the HAS (French National Authority for Health), appears to be the “Clinical Practice Guidelines” method [22]. An analysis and critical synthesis of the scientific literature were conducted according to the principles of critical reading to attribute a level of scientific proof to each article based on the classifications proposed by the HAS [23] (Table 1).

**Table 1:** Recommendation grading

Level of scientific proof provided by the literature (for clinical studies)	Recommendation grading
*Level 1* High-power randomised comparative studies Meta-analysis of randomised comparative studies Decision analysis based on well-conducted studies	*A* Scientific proof established
*Level 2* Low-power randomised comparative studies Well-conducted non-randomised comparative studies Cohort studies	*B* Scientific proof presumed
*Level 3* Case–control studies	*C* Low level of proof
*Level 4* Comparative studies with major bias Retrospective studies Case series Descriptive epidemiological studies (cross-sectional, longitudinal)	

No randomized comparative studies have been conducted on occupational risk factors in the workplace. However, there have been several “well-conducted” studies, taking into account confounding factors and potential dose–response relationships, that report concordant results. We first selected meta-analyses or systematic reviews of well-conducted cohorts offering level 2 scientific proof, then cohort studies offering level 2 scientific proof and, finally, case–control studies offering level 3 scientific proof. The associated recommendation grades are illustrated in Table 1.

Due to a lack of available studies, certain recommendations are based on expert consensus within the framework of a working group after consultation with a reading group. Composition of working group (24 members) and reading group (72 members) is presented in Additional file 1 along with the consulted databases and keywords used.

The scientific rationale used to elaborate the recommendations, established by the project coordinator was forwarded to all members of the working group. The working group then amended and/or completed the list of recommendations to draft a new version. This new version was sent to the reading group. The comments offered by the reading group were analyzed by the working group, which then modified the rationale based on certain remarks before drafting a final version of the recommendations. The final version of the rationale and recommendations, together with the process implemented for their production, was then analyzed by the HAS Committee for health care strategies and the HAS College. Because no humans were involved in this study, no Ethics Committee or Institutional Review Board approval was necessary. For the same reason, no written informed consent was necessary.

## Results

**Fig. 1:**
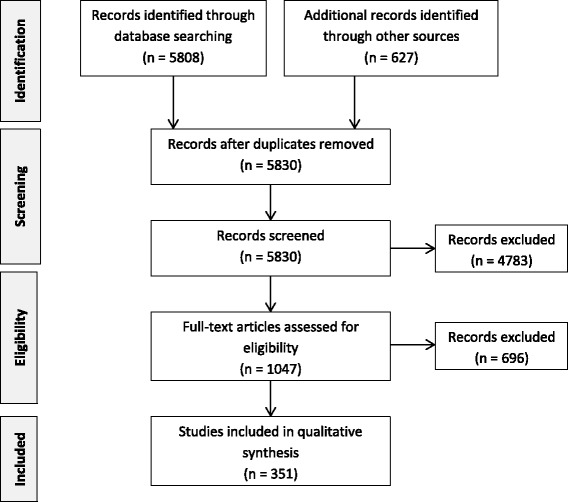
Flow diagram

The flow chart of the search strategy is presented in Fig. 1. The results of our analysis of the scientific literature on carcinogenic to humans (group 1) with sufficient evidence in humans regarding lung cancer were summarized in Additional file 2 with regard to the possible existence of the following:

- a dose–response relationship in BPC,
- a threshold of carcinogenic effects (i.e., a threshold above which a carcinogenic effect can be detected) for mean exposure intensity, peak exposure values, exposure duration or cumulative exposure.
- modeling of the incidence of BPC based on co-exposures.

Only one large-scale randomized study (*n* = 53,456), the NLST, which was conducted in the United States with subjects aged from 55 to 74 years who were either active or former smokers (having stopped within the past 15 years) with a consumption of 30 pack-years or more, demonstrated the efficacy of low-dose chest CT scan screening; this study reported a significant 20% reduction in mortality by BPC and a 6.7% reduction in overall mortality compared to screening by chest X-ray alone [11]. Other randomized clinical trials reported results on mortality by BPC: three with non-significant results (DANTE study, DLCST study and MILD study) [24–26]. Two other randomized clinical trials were ongoing (NELSON, UKLS) [27, 28]. No randomized clinical trial enabled the evaluation of the reduction in mortality through BPC screening in the specific population of subjects professionally exposed to lung carcinogens. Based on the literature published on the risk of bronchopulmonary cancer associated with tobacco consumption, the relative risk of bronchopulmonary cancer for smokers with a consumption of over 30 pack years (PY) is estimated to be equal to or in excess of 30; for smokers with a consumption between 20 and 30 PY, it is estimated at 20, and for those with a consumption between 10 and 20 PY, it is estimated at 10 [29]. In former smokers who have stopped smoking within the past 15 years, the relative risk of BPC is estimated to be five [29]. No occupational carcinogen considered alone obtains the same level of risk in non-smokers (except for arsenic and BCME).

In order to propose screening in accordance with the NLST criteria and to account for occupational risk factors, we needed to consider the types of occupational exposures among smokers and former smokers that had a risk level at least equivalent to the NLST results. Hence, in Table 2, the working group proposed an estimation of the relative risk (RR) of each occupational lung carcinogen and tobacco, based on the available data in the literature. For all carcinogens included in the rationale, we assumed that the cumulative effect of two risk factors on the risk of bronchopulmonary cancer was multiplied. For example, in a subject with a risk level of 30, adopting a multiplicative model for former smokers who have not smoked for over 15 years and have been exposed to soot, the risk of bronchopulmonary cancer was estimated to be 5 × 2 = 10. We considered the fact that for subjects with a high risk of bronchopulmonary cancer in the NLST study, the RR was approximately 30. In Table 2, the following is provided:

**Table 2:** Estimation of BPC risk associated with occupational risk factors and tobacco consumption (Expert consensus)

	Relative risk according to exposure to carcinogens	Estimated risk level				
		Non-smokers	Ex-smokers ≥ 15 years	Smokers		
Agents, situations or processes				<20 PY	20 – 29 PY	≥30 PY
Tobacco		1	5	10	20	***30***
Asbestos – intermediate cumulative level < 10 years	1.5	1.5	7.5	15	***30***	***45***
Asbestos – intermediate cumulative level ≥ 10 years	2	2	10	20	***40***	**60**
Asbestos – high cumulative level < 5 years	2.5	2.5	12.5	25	***50***	**75**
Asbestos – high cumulative level ≥ 5 years	3	3	15	***30***	**60**	**90**
Asbestosis	3	3	15	***30***	**60**	**90**
Pleural plaques	2	2	10	20	***40***	**60**
Crystalline silica	1.5	1.5	7.5	15	***30***	***45***
Silicosis	2	2	10	20	***40***	**60**
Diesel exhaust fumes – intermediate level	1.5	1.5	7.5	15	***30***	***45***
Diesel exhaust fumes – high level	2	2	10	20	***40***	**60**
Aluminium production	2	2	10	20	***40***	**60**
Coal gasification	2	2	10	20	***40***	**60**
Coal tar pitch	2	2	10	20	***40***	**60**
Coke production	2	2	10	20	***40***	**60**
Soot	2	2	10	20	***40***	**60**
X-rays and gamma rays	2	2	10	20	***40***	**60**
Radon	2	2	10	20	***40***	**60**
Iron ore mines	2	2	10	20	***40***	**60**
Plutonium	10	10	***50***	**100**	**200**	**300**
Iron and steel foundry	1.5	1.5	7.5	15	***30***	***45***
Painting profession	2	2	10	20	***40***	**60**
Rubber production	2	2	10	20	***40***	**60**
Arsenic and its compounds	5	5	25	***50***	**100**	**150**
Nickel compounds	2	2	10	20	***40***	**60**
Chromium(VI) compounds	2	2	10	20	***40***	**60**
Beryllium	2	2	10	20	***40***	**60**
Cadmium and its compounds	2	2	10	20	***40***	**60**
Bis(chloromethyl) ether; Chloromethyl methyl ether	10	10	***50***	**100**	**200**	**300**
Metal cobalt associated with tungsten carbide	2	2	10	20	***40***	**60**

These RR estimations were retained by the working group based on data from the literature and on the hypothesis of the multiplicative joint effect of a carcinogenic agent and tobacco

normal: risk level < 30; ***italic bold: 30 < risk level > 60***; **bold underlined: risk level ≥ 60**

- in normal topography – subjects for whom the estimated relative risk of bronchopulmonary cancer is lower than that of NLST subjects;
- in bold italic – subjects for whom the estimated relative risk is close to that of the NLST subjects, i.e., between 30 and 60; and
- in bold underlined – subjects for whom the estimated relative risk is higher than that of the NLST subjects, i.e., equal to or above 60.

In the review of the literature focusing on occupational risk factors for BPC, the reported studies included subjects with highly varied periods of occupational exposure, ranging from less than 1 year to the total duration of their professional career. Hence, and from a pragmatic point of view, the mean risks calculated in Table 2 apply to subjects with an exposure duration of 10 years.

Asbestos exposure is defined based on the report provided by the jury of the 1999 French consensus conference on the follow-up of asbestos-exposed workers [30].

- High cumulative exposure: Confirmed, high level and continued exposure of a duration equal to or in excess of 1 year. For example, professional activities in manufacturing or in the transformation of materials including asbestos and their equivalents when working on materials or equipment likely to discharge asbestos fibers (e.g., fireproofing, naval construction); Confirmed, high level and discontinued exposure of a duration equal to or in excess of 10 years (e.g., mechanics/machine operators on heavy goods vehicle brake systems, cutting of asbestos cement).
- Intermediate cumulative exposure: All other documented situations with significant occupational exposure. The majority of these situations involve working with materials or equipment likely to discharge asbestos fibers.

From a practical point of view, high-level exposure corresponds roughly to a fiber concentration above ten fibers/ml (8 h Time-Weighted Average (TWA)) and an intermediate level to a fiber concentration above 0.1 f/ml (8 h TWA).

## Discussion

Simulation studies have been conducted since the publication of the NLST results in an attempt to better define the groups of subjects who may benefit from chest CT scan screening. These studies demonstrated that the higher the risk of bronchopulmonary cancer among subjects included in a screening procedure, the more the benefit-risk balance leaned towards benefit [31–35]. Among limitations of low-dose CT scan, we found false-positives and overdiagnosis [36] but these limitations could be reduced using appropriate reading and follow-up protocol of lung nodules [37]. Another limitation of screening by low-dose chest CT scan is the radiation exposure due to the repetition of scan [36].In such conditions, and given the lack of data on BPC screening in occupationally exposed workers, the working group suggests a strictly controlled experiment on BPC screening in high-risk subjects, i.e., subjects with an occupational exposure to lung carcinogens that indicates a high risk of BPC.

The high-risk population as defined by the working group is presented in Table 3

**Table 3:** Definition of high-risk subjects for BPC (aged from 55 to 74 years) (Expert consensus)

Occupational pollutant	Cumulative level of exposure or disease	Cumulative exposure duration	Active or former tobacco consumption dating back less than 15 years
Asbestos	Intermediate^b^	≥10 years	≥30 PY
	High	<5 years	≥30 PY
	High	≥5 years	≥20 PY
	Asbestosis		≥20 PY
	Pleural plaques		≥30 PY
Other carcinogenic agents^a^		≥10 years	≥30 PY
Co-exposure			
2 carcinogenic agents		≥10 years	≥20 PY
≥ 3 carcinogenic agents		≥10 years	≥10 PY

^a^aluminium production, coal gasification, coal tar pitch, coke production, X-rays and gamma rays, radon, iron ore mines, plutonium, steel foundries, the painting profession, rubber production, chromium(VI) compounds, beryllium, cadmium and its compounds, bis(chloromethyl) ether, chloromethyl methyl ether, metal cobalt with tungsten carbide

Special cases: Crystalline silica (silicosis is necessary to integrate the high-risk group for BPC, independently of the duration of exposure); diesel engine exhaust fumes (a high level of exposure defined by employment in underground mines, tunnel construction or underground mine maintenance is necessary to integrate the high-risk group for BPC)

^b^In the sense of the jury of the 1999 french consensus conference on the follow-up of asbestos-exposed workers

High exposure: Confirmed, high and continued exposure of a duration equal to or in excess of one year; examples: professional activities in the manufacture or transformation of materials including asbestos and their equivalents during intervention on materials or equipment likely to discharge asbestos fibres (e.g.: fireproofing, naval construction); Confirmed, high and discontinued exposure of a duration equal to or in excess of 10 years (e.g.: mechanics/machine operators on heavy goods vehicle brake systems, cutting of asbestos cement)

Intermediate exposure: All other documented occupational significant exposure situations. The majority of these situations involve intevention on materials or equipment likely to discharge asbestos fibres

All other risk situations are to be considered on an individual basis by the responsible health care center.

Hence, the working group recommends the following:

- the implementation of a screening experiment for bronchopulmonary cancer in subjects occupationally exposed or having been occupationally exposed to lung carcinogens confirmed as high-risk factors for BPC using low-dose chest CT scan (Expert consensus). This experiment, which will be conducted in reference healthcare centers, should enable the evaluation of the feasibility of such screening. Moreover, little studies have evaluated the cost-effectiveness ratio of the BPC screnning. In NLST this ratio was assessed at 52 000USD per year gained [38]. Thus, experiment will be evaluated this cost-effectiveness ratio.
- the assessment of individual bronchopulmonary cancer risk to determine the most suitable medico-professional surveillance for each worker. This assessment must be based on professional and clinical history and it should take into account all risk factors, including confirmed occupational lung carcinogens (IARC group 1) associated or not with tobacco consumption.
- to encourage or to lead smokers, regardless of eligibility for screening, to benefit from guidance on how to stop smoking (Expert consensus)

Other than the screening experiment, the experts do not recommend screening by low-dose chest CT scan among workers currently or formerly occupationally exposed to lung carcinogens (Expert consensus) (indeed, given the lack of specific studies on this population and of appropriately organized structures, the conditions were considered insufficient to translate the results of the North American NLST study to this population (Expert consensus)).

Following the conclusions of the working group, a recent review of the literature on the effectiveness, acceptability and safety of lung cancer screening with LDCT in subjects highly exposed to tobacco determined, in regard to the lack of strong scientific evidence, that LDCT screening should not be recommended in subjects with high exposures to tobacco [39].

## Conclusions

The working group’s proposal on the need to implement a screening experiment for bronchopulmonary cancer, in subjects occupationally exposed or having been occupationally exposed to lung carcinogens confirmed as high-risk factors for BPC with low-dose chest CT scan, is in line with previously internationally published recommendations and expert opinions [12–21].

## Additional files

Additional file 1: Composition of working group and reading group, consulted databases and keywords used. (DOCX 15 kb)
Additional file 2: Level of risk of BPC associated with occupational risks. (DOCX 123 kb)

## Abbreviations

BCME: Bis(chloromethyl) ether and chloromethyl methyl ether
BPC: Bronchopulmonary cancer
CNAM-TS: French salaried workers’ health insurance fund
DGS: Directorate General for Health
DGT: Directorate General for Labour
HAS: French National Authority for Health
IARC: the International Agency for Research on Cancer
INCa: French National Cancer Institute
INRS: National Research and Safety Institute
InVS: French Institute for Public Health Surveillance
NLST: the National Lung Screnning trial
PY: Pack years
RR: Relative risk
SFMT: French Society of Occupational Medicine
SFR: French Society of Radiology
SPLF: French-Speaking Society of Pneumology
TWA: Time Weighted Average
WHO: World Health Organisation

## Footnotes

1 List of Classifications by cancer sites with sufficient or limited evidence in humans, Volumes 1 to 112, IARC
