# Supplementary material for: Medical follow-up of workers exposed to lung carcinogens: French evidence-based and pragmatic recommendations
Source: BMC Public Health. 2017 Feb 14;17:191. doi: 10.1186/s12889-017-4114-1 (PMC5307847; doi:10.1186/s12889-017-4114-1)
Supplement: Additional file 1: — Composition of working group and reading group, consulted databases and keywords used. (DOCX 15 kb) [file 12889_2017_4114_MOESM1_ESM.docx]

Consulted databases

The consulted databases included the following: Medline (National Library of Medicine, USA), Cochrane Library (Wiley Interscience, USA), Pascal - Institut national de l’information scientifique et technique (National scientific and technical information institute) France, National Guideline Clearinghouse (Agency for Healthcare Research and Quality, USA), and Guidelines Finder (National Library for Health, USA). The following websites were also consulted: IARC, INRS (National Research and Safety Institute for the prevention of accidents in the workplace and occupational diseases), InVS (French Institute for Public Health Surveillance), HAS, Lemanissier medical library, National Institute for Health and Clinical Excellence, Scottish Intercollegiate Guidelines Network, National Institute for Occupational Safety and Health and the websites of learned societies involved in the project. Other bibliographic sources were also consulted: bibliographic references quoted in the analyzed articles, the National Toxicology Program classification of carcinogenic chemical substances, and the European Union classification of dangerous substances.

Keywords

With regard to occupational risk factors, we exclusively selected publications in French or in English dating from 1990 to June 1, 2015, and used the following keywords: for BPC: "lung", "pulmonary", "bronchopulmonary", and "bronchial" each systematically associated with "neoplasm", "cancer", "carcinoma", "blastoma" and "tumor/tumour"; for occupational exposure: "Occupational Exposure", "Occupational Diseases", "occupational", "work-related", and "worker"; and for each carcinogenic agent: "asbestos", "silica", "aluminum", "coal", "diesel", "coke", "soot", "painter", "rubber", "arsenic", "nickel", "chromium", "cadmium", "beryllium", "bis(chloromethyl) ether" and "chloromethyl methyl ether". With regard to BPC, we conducted an update of the literature reported after the publication of two meta-analyses, i.e., for the years 2014 and 2015, using the keywords: "lung neoplasms" and "screening". When we studied the risk of bronchopulmonary cancer associated with tobacco consumption, considering the existing literature on the subject and changes in cigarette composition and tobacco consumption trends, we focused on publications issued since 2000, using, in addition to those associated with BPC, the following keywords: "lung neoplasms", "risk" and "smoking".

Working group

A working group composed of 24 members was created. It comprised 4 members from the SFMT (French Society of Occupational Medicine); 3 members from the SPLF (French-Speaking Society of Pneumology); 2 members from the SFR (French Society of Radiology); 1 member from the INCa (French National Cancer Institute); 2 engineering specialists in occupational toxicity and preventive measures against pulmonary pollutants in the workplace; 1 member from the InVS; 2 inter-company occupational physicians; 2 general practitioners; 1 epidemiologist; 1 public health physician specializing in pneumology; 1 occupational health nurse; 1 medical officer from the CNAM-TS (French salaried workers' health insurance fund); 1 member from the Ligue Nationale Contre le Cancer; 1 HAS project leader; and 1 project coordinator.

Reading group

This multidisciplinary reading group comprised 72 healthcare professionals and other addressees included due to their interest in the broached subject. The reading group comprised the following: 15 occupational physicians, 21 pneumologists, 7 radiologists, 10 general practitioners, 5 occupational health inspectors, 3 occupational health nurses, 1 medical officer, 1 engineer, 1 health economist, 2 members of the DGT (Directorate General for Labor), 1 member of the INRS, 1 member of the INCa, 1 member of the DGS (Directorate General for Health), 1 former medical inspector, and 2 social partners.
