# Supplementary material for: Medical follow-up of workers exposed to lung carcinogens: French evidence-based and pragmatic recommendations
Source: BMC Public Health. 2017 Feb 14;17:191. doi: 10.1186/s12889-017-4114-1 (PMC5307847; doi:10.1186/s12889-017-4114-1)
Supplement: Additional file 2: — Level of risk of BPC associated with occupational risks. (DOCX 123 kb) [file 12889_2017_4114_MOESM2_ESM.docx]

Additional file 2: Level of risk of BPC associated with occupational risks

| **Agents, situations or processes** | **Dose-response relationship and exposure duration** | **Co-exposure / Associated diseases** |
| --- | --- | --- |
| **Asbestos**  **Mean model***  **Maximalist model*** | **YES (level of proof 2)**  Variation of relative risk (RR) between 0.1 and 4% per f/ml.year (level of proof 2). Taking the value of 1.0% as the relative risk variation as proposed by the Inserm's collective expertise group, (1) for exposure of:  10 f/ml.years the RR is 1.10  25 f/ml.years the RR is 1.25  50 f/ml.years the RR is 1.50  Relative risk variation of 4% per f/ml.year (2), for exposure of:  10 f/ml.years the RR is 1.40  25 f/ml.years the RR is 2  50 f/ml.years the RR is 3 | **Tobacco**  Multiplicative joint effect: tobacco risk × asbestos risk (level of proof 2) (3-5)  **Other carcinogenic agents**  Not established  **Asbestosis**  Risk of BPC multiplied by 2 (6, 7) to 4 (8) (level of proof 2)  **Pleural plaques**  Risk of BPC multiplied by 2 (9) (level of proof 2) |
| **Crystalline silica**  **Mean model**  **Maximalist model** | **YES (level of proof 2)**  RR between 1 and 1.5 for cumulative exposure > 2 mg/m^3^×years (level of proof 2) (10, 11)  Exposure ranging from 1 to 2 mg/m^3^×years, results are heterogeneous.  1.0 mg/m^3^×years: RR = 1.2 (level of proof 2) (10)  6.0 mg/m^3^×years: RR = 1.8 (level of proof 2) (10) | **Tobacco**  Multiplicative joint effect: tobacco risk × silica risk (level of proof 2) (12-15)  **Radon**  Insufficient data  **Other carcinogenic agents**  Not established  **Silicosis**  RR in excess of 2 (16) (level of proof 2) |
| **Diesel engine exhaust fumes**  **Mean model**  **Maximalist model** | **YES (level of proof 2)**  RR between 1.17 and 2.44 for exposure durations from 10 to 30 years (level of proof 2) (17, 18)  For cumulative exposure to elemental carbon (19)  > 30 µg/m^3^.years: RR between 1 and 1.5  > 500 µg/m^3^.years: RR > 2 (level of proof 2)  Exposure < 10 years: RR = 1.4 (20) (level of proof 2)  Exposure ≥ 20 years: RR = 2.4 (21) (level of proof 2)  Exposure to elemental carbon  30.9-71.7 µg/m^3^.years: RR = 1.3 (22) (level of proof 2)  ≥ 536 µg/m^3^.years: RR = 2.8 (22) (level of proof 3) | **Tobacco**  Heterogeneous data  **Other carcinogenic agents**  Not established |
| **Aluminium production**  **Mean model**  **Maximalist model** | **YES (level of proof 2)**  Cumulative exposure to BaP** > 80 µg/m^3^.years and to BSM** > 2.0 mg/m^3^.years: RR between 1.5 and 2 (level of proof 2) (23-25)  Exposure to BaP expressed in µg/m3.years (26)  20 – 40: RR = 2 (level of proof 2)  ≥ 320: RR = 3 (level of proof 2)  Exposure to BSM expressed in µg/m3.years (26)  2.0 – 4.0: RR = 1.5 (level of proof 2)  ≥ 32.0: RR = 2 (level of proof 2)  Duration of employment between 10 and 20 years: RR = 5 (27) (level of proof 2) | **Tobacco**  Insufficient data  **Other carcinogenic agents**  Not established |
| **Coal gasification** | Insufficient data on the dose-response relationship  RR associated with exposure is between 1.5 and 3 (level of proof 2) (28-30) | **Tobacco**  Not established  **Other carcinogenic agents**  Not established |
| **Coal tar pitch**  **Mean model**  **Maximalist model** | Heterogeneous data on the dose-response relationship  RR associated with exposure is between 1.5 and 5 (level of proof 2) (31)  "Moderate exposure" group: RR = 10 (32) (level of proof 3) | **Tobacco**  Insufficient data  **Other carcinogenic agents**  Not established |
| **Coke production**  **Mean model**  **Maximalist model** | **YES (level of proof 2)**  Cumulative exposure to BaP, exposure > 30 µg. m^3^.years: RR between 1.5 and 2 (level of proof 2) (33)  Exposure duration > 5 years, RR between 1.5 and 2 (level of proof 2) (33)  Between 15 and 19 years, RR = 3 (34) (level of proof 2)  Exposure duration > 5 years, RR = 2 (level of proof 2) (33)  Exposure to BaP > 30 µg/m^3^.years: RR = 1.5 (33) (level of proof 2) | **Tobacco**  Not established  **Other carcinogenic agents**  Not established |
| **Soot** | Insufficient data on the dose-response relationship  RR associated with exposure is in excess of 2 (level of proof 2) | **Tobacco**  Not established  **Other carcinogenic agents**  Not established |
| **X-rays and gamma rays**  **Mean model**  **Maximalist model** | **YES with exposure duration (level of proof 2)**  RR > 3 for an exposure duration > 20 years (level of proof 2) (35)  RR > 3 for an exposure duration > 20 years (level of proof 2) (35) | **Tobacco**  Not established  **Other carcinogenic agents**  Not established |
| **Radon**  **Mean model** | **YES (level of proof 2)**  Excess absolute risk of lung cancer throughout the entire life associated with radon exposure is 5 × 10^-4^ per working level month (14 × 10^-5^ per mJh/m3) (level of proof 2) (36) | **Tobacco**  Multiplicative joint effect: tobacco risk × radon risk (level of proof 3)  **Silica**  Insufficient data  **Other carcinogenic agents**  Not established |
| **Iron ore mines** | **YES (level of proof 2)**  Exposure duration between 5 and 14 years, RR between 1.5 and 2 (level of proof 2) (37)  Exposure duration > 15 years, RR > 2 (level of proof 2) (37)  Exposure duration > 20 years, RR between 5 and 7 (38) | **Tobacco**  Not established  **Other carcinogenic agents**  Not established |
| **Plutonium** | Heterogeneous data  RR = 8 for men / RR = 25 for women (one study (39)) | **Tobacco**  Insufficient data  **Other carcinogenic agents**  Not established |
| **Iron and steel foundry** | Heterogeneous results on the duration-response relationship  Exposure duration > 30 years, RR > 2 (level of proof 3) (one study (40))  RR associated with exposure is between 1 and 2 (level of proof 2) | **Tobacco**  Not established  **Other carcinogenic agents**  Not established |
| **Painting profession** | **YES (level of proof 2)**  Exposure duration > 10 years, RR between 1.5 and 2 (level of proof 2) (41)  Exposure duration > 20 years, RR > 2 (level of proof 2) (41) | **Tobacco**  Not established  **Other carcinogenic agents**  Not established |
| **Rubber production** | Heterogeneous data on the dose-response relationship  RR associated with exposure between 1.5 and 2 (level of proof 2) | **Tobacco**  Not established  **Other carcinogenic agents**  Not established |
| **Arsenic and its compounds**  **Mean model**  **Maximalist model** | **YES (level of proof 2)**  RR associated with exposure > 2 (level of proof 2)  Highest exposure groups RR > 5 (level of proof 2) (42, 43)  Cumulative arsenic exposure ≥ 100 mg/m^3^× years: RR = 9 (level of proof 3) (42) | **Tobacco**  Insufficient data  **Cadmium**  Insufficient data  **Other carcinogenic agents**  Not established |
| **Nickel compounds**  **Mean model**  **Maximalist model** | **YES (level of proof 2)**  Cumulative soluble nickel exposure ≥ 2 mg/m^3^×years, RR > 2 (level of proof 2) (44-46)  Cumulative exposure to nickel oxide ≥ 0.13 mg/m^3^×years, RR > 2 (level of proof 2) (44-46)  Heterogeneous duration-response data  Exposure duration > 20 years, RR between 1 and 1.5 (level of proof 3) (two studies (47, 48))  Water-soluble nickel, median exposure 4.93 mg/m^3^×years: OR = 4 (level of proof 3) (45)  Nickel sulphur, median exposure 1.43 mg/m^3^×years: OR = 3 (level of proof 3) (45)  Nickel oxide exposure, median exposure 0.36 mg/m^3^×years: OR = 3 (level of proof 3) (45)  Metallic nickel, median exposure 2.32 mg/m^3^×years: OR = 2 (level of proof 3) (45) | **Tobacco**  Heterogeneous data  **Other carcinogenic agents**  Not established |
| **Chromium(VI) compounds**  **Mean model**  **Maximalist model** | **YES (level of proof 2)**  RR associated with exposure is between 1.5 and 2 (level of proof 2)  Cumulative CrO_3_ exposure between 4.45 and 29 mg/m^3^-years: RR for employment ≥ 30 years: RR = 2 (49) | **Tobacco**  Insufficient data  **Other carcinogenic agents**  Not established |
| **Beryllium** | Heterogeneous results  Cumulative dose of 2 µg/m^3^ per day RR > 2 (level of proof 2) (50)  Cumulative dose of 8 µg/m3 per day RR> 5 (level of proof 2) (50)  Exposure duration > 25 years, RR > 2 (level of proof 2) (51)  Cumulative dose between 8.0 and 12.0 µg/m^3^ per day RR = 7 (50)  Exposure duration ≥ 35 years: RR = 4 (51) | **Tobacco**  Insufficient data  **Other carcinogenic agents**  Not established  **Acute berylliosis**  SMR > 2 (level of proof 2)  **Chronic berylliosis**  Absence of excess risk (level of proof 2) |
| **Cadmium and its compounds** | **YES (level of proof 2)**  Cumulative exposure > 10 mg/m^3^ × years: RR > 2 (level of proof 2) (43) | **Tobacco**  Insufficient data  **Arsenic**  Insufficient data  **Other carcinogenic agents**  Not established |
| **Bis(chloromethyl) ether Chloromethyl methyl ether**  **Mean model**  **Maximalist model** | **YES (level of proof 2)**  RR associated with exposure > 2 (level of proof 2) (52, 53)  Median cumulative exposure of 24.0 score-years: RR = 40.0 (52) (exposure is quantified by means of a score) | **Tobacco**  Not established  **Other carcinogenic agents**  Not established |
| **Metal cobalt associated with tungsten carbide** | Insufficient data  Exposure duration > 10 years: OR > 2 (level of proof 3) (54)  Unweighted cumulative dose (in months × level of exposure) > 299: OR = 4 (level of proof 3) (54) | **Tobacco**  Insufficient data  **Other carcinogenic agents**  Not established |
| ***The maximalist model is the highest risk level found in the literature, the mean level is the most frequently found risk level in the literature.**  **BaP: benzo(a)pyrene; BSM: benzene soluble matter | | |

1. Expertise collective. Effets sur la santé des principaux types d’exposition à l’amiante. Paris Inserm; 1997. 450 p.

2. Hodgson JT, Darnton A. The quantitative risks of mesothelioma and lung cancer in relation to asbestos exposure. The Annals of occupational hygiene. 2000;44(8):565-601.

3. Lee PN. Relation between exposure to asbestos and smoking jointly and the risk of lung cancer. Occupational and environmental medicine. 2001;58(3):145-53.

4. Liddell FD. The interaction of asbestos and smoking in lung cancer. The Annals of occupational hygiene. 2001;45(5):341-56.

5. Wraith D, Mengersen K. Assessing the combined effect of asbestos exposure and smoking on lung cancer: a Bayesian approach. Statistics in medicine. 2007;26(5):1150-69.

6. Suivi post-professionnel après exposition à l'amiante - Audition publique. Haute Autorité de Santé, 2011.

7. Markowitz SB, Levin SM, Miller A, Morabia A. Asbestos, asbestosis, smoking, and lung cancer. New findings from the North American insulator cohort. American journal of respiratory and critical care medicine. 2013;188(1):90-6.

8. Hughes JM, Weill H. Asbestosis as a precursor of asbestos related lung cancer: results of a prospective mortality study. British journal of industrial medicine. 1991;48(4):229-33.

9. Pairon JC, Andujar P, Rinaldo M, Ameille J, Brochard P, Chamming's S, et al. Asbestos Exposure, Pleural Plaques and the Risk of Death from Lung Cancer. American journal of respiratory and critical care medicine. 2014.

10. Lacasse Y, Martin S, Gagne D, Lakhal L. Dose-response meta-analysis of silica and lung cancer. Cancer causes & control : CCC. 2009;20(6):925-33.

11. Steenland K, Mannetje A, Boffetta P, Stayner L, Attfield M, Chen J, et al. Pooled exposure-response analyses and risk assessment for lung cancer in 10 cohorts of silica-exposed workers: an IARC multicentre study. Cancer causes & control : CCC. 2001;12(9):773-84.

12. De Matteis S, Consonni D, Lubin JH, Tucker M, Peters S, Vermeulen R, et al. Impact of occupational carcinogens on lung cancer risk in a general population. International journal of epidemiology. 2012;41(3):711-21.

13. Liu Y, Steenland K, Rong Y, Hnizdo E, Huang X, Zhang H, et al. Exposure-response analysis and risk assessment for lung cancer in relationship to silica exposure: a 44-year cohort study of 34,018 workers. American journal of epidemiology. 2013;178(9):1424-33.

14. Tse LA, Yu IT, Qiu H, Leung CC. Joint effects of smoking and silicosis on diseases to the lungs. PloS one. 2014;9(8):e104494.

15. Vida S, Pintos J, Parent ME, Lavoue J, Siemiatycki J. Occupational exposure to silica and lung cancer: pooled analysis of two case-control studies in Montreal, Canada. Cancer epidemiology, biomarkers & prevention : a publication of the American Association for Cancer Research, cosponsored by the American Society of Preventive Oncology. 2010;19(6):1602-11.

16. IARC Working Group on the Evaluation of Carcinogenic Risks to Humans. Arsenic, Metals, Fibres, and Dusts Vol 100C. Lyon: IARC; 2012.

17. Olsson AC, Gustavsson P, Kromhout H, Peters S, Vermeulen R, Bruske I, et al. Exposure to diesel motor exhaust and lung cancer risk in a pooled analysis from case-control studies in Europe and Canada. American journal of respiratory and critical care medicine. 2011;183(7):941-8.

18. Swanson GM, Lin CS, Burns PB. Diversity in the association between occupation and lung cancer among black and white men. Cancer epidemiology, biomarkers & prevention : a publication of the American Association for Cancer Research, cosponsored by the American Society of Preventive Oncology. 1993;2(4):313-20.

19. Vermeulen R, Silverman DT, Garshick E, Vlaanderen J, Portengen L, Steenland K. Exposure-response estimates for diesel engine exhaust and lung cancer mortality based on data from three occupational cohorts. Environmental health perspectives. 2014;122(2):172-7.

20. Lipsett M, Campleman S. Occupational exposure to diesel exhaust and lung cancer: a meta-analysis. American journal of public health. 1999;89(7):1009-17.

21. Bhatia R, Lopipero P, Smith AH. Diesel exhaust exposure and lung cancer. Epidemiology (Cambridge, Mass). 1998;9(1):84-91.

22. Vermeulen R, Portengen L, Silverman DT, Garshick E, Steenland K. Meta-analysis of lung cancer risk from exposure to diesel exhaust: vermeulen et Al. Respond. Environmental health perspectives. 2014;122(9):A230-1.

23. Armstrong BG, Gibbs G. Exposure-response relationship between lung cancer and polycyclic aromatic hydrocarbons (PAHs). Occupational and environmental medicine. 2009;66(11):740-6.

24. Gibbs GW, Sevigny M. Mortality and cancer experience of Quebec aluminum reduction plant workers. Part 3: monitoring the mortality of workers first employed after January 1, 1950. Journal of occupational and environmental medicine / American College of Occupational and Environmental Medicine. 2007;49(11):1269-87.

25. Spinelli JJ, Demers PA, Le ND, Friesen MD, Lorenzi MF, Fang R, et al. Cancer risk in aluminum reduction plant workers (Canada). Cancer causes & control : CCC. 2006;17(7):939-48.

26. Gibbs GW, Armstrong B, Sevigny M. Mortality and cancer experience of Quebec aluminum reduction plant workers. Part 2: mortality of three cohorts hired on or before january 1, 1951. Journal of occupational and environmental medicine / American College of Occupational and Environmental Medicine. 2007;49(10):1105-23.

27. Sim MR, Del Monaco A, Hoving JL, Macfarlane E, McKenzie D, Benke G, et al. Mortality and cancer incidence in workers in two Australian prebake aluminium smelters. Occupational and environmental medicine. 2009;66(7):464-70.

28. Berger J, Manz A. Cancer of the stomach and the colon-rectum among workers in a coke gas plant. American journal of industrial medicine. 1992;22(6):825-34.

29. Doll R, Vessey MP, Beasley RW, Buckley AR, Fear EC, Fisher RE, et al. Mortality of gasworkers - final report of a prospective study. British journal of industrial medicine. 1972;29(4):394-406.

30. Martin JC, Imbernon E, Goldberg M, Chevalier A, Bonenfant S. Occupational risk factors for lung cancer in the French electricity and gas industry: a case-control survey nested in a cohort of active employees. American journal of epidemiology. 2000;151(9):902-12.

31. IARC Working Group on the Evaluation of Carcinogenic Risks to Humans. Chemical agents and related occupations Vol 100F. Lyon: IARC; 2012. 628 p.

32. Kauppinen T, Heikkila P, Partanen T, Virtanen SV, Pukkala E, Ylostalo P, et al. Mortality and cancer incidence of workers in Finnish road paving companies. American journal of industrial medicine. 2003;43(1):49-57.

33. Miller BG, Doust E, Cherrie JW, Hurley JF. Lung cancer mortality and exposure to polycyclic aromatic hydrocarbons in British coke oven workers. BMC public health. 2013;13:962.

34. Costantino JP, Redmond CK, Bearden A. Occupationally related cancer risk among coke oven workers: 30 years of follow-up. Journal of occupational and environmental medicine / American College of Occupational and Environmental Medicine. 1995;37(5):597-604.

35. Cardis E, Vrijheid M, Blettner M, Gilbert E, Hakama M, Hill C, et al. The 15-Country Collaborative Study of Cancer Risk among Radiation Workers in the Nuclear Industry: estimates of radiation-related cancer risks. Radiation research. 2007;167(4):396-416.

36. Tirmarche M, Harrison JD, Laurier D, Paquet F, Blanchardon E, Marsh JW. ICRP Publication 115. Lung cancer risk from radon and progeny and statement on radon. Annals of the ICRP. 2010;40(1):1-64.

37. Bergdahl IA, Jonsson H, Eriksson K, Damber L, JÄRvholm B. Lung cancer and exposure to quartz and diesel exhaust in Swedish iron ore miners with concurrent exposure to radon (English). Occupational and environmental medicine (London). 2010;67(8):513-8.

38. Damber L, Larsson LG. Underground mining, smoking, and lung cancer: a case-control study in the iron ore municipalities in northern Sweden. Journal of the National Cancer Institute. 1985;74(6):1207-13.

39. Gilbert ES, Sokolnikov ME, Preston DL, Schonfeld SJ, Schadilov AE, Vasilenko EK, et al. Lung cancer risks from plutonium: an updated analysis of data from the Mayak worker cohort. Radiation research. 2013;179(3):332-42.

40. Becher H, Jedrychowski W, Flak E, Gomola K, Wahrendorf J. Lung cancer, smoking, and employment in foundries. Scandinavian journal of work, environment & health. 1989;15(1):38-42.

41. Guha N, Merletti F, Steenland NK, Altieri A, Cogliano V, Straif K. Lung cancer risk in painters: a meta-analysis. Ciencia & saude coletiva. 2011;16(8):3613-32.

42. Jarup L, Pershagen G. Arsenic exposure, smoking, and lung cancer in smelter workers--a case-control study. American journal of epidemiology. 1991;134(6):545-51.

43. Park RM, Stayner LT, Petersen MR, Finley-Couch M, Hornung R, Rice C. Cadmium and lung cancer mortality accounting for simultaneous arsenic exposure (English). Occupational and environmental medicine (London). 2012;69(5):303-9.

44. Andersen A, Berge SR, Engeland A, Norseth T. Exposure to nickel compounds and smoking in relation to incidence of lung and nasal cancer among nickel refinery workers. Occupational and environmental medicine. 1996;53(10):708-13.

45. Grimsrud TK, Berge SR, Haldorsen T, Andersen A. Exposure to different forms of nickel and risk of lung cancer. American journal of epidemiology. 2002;156(12):1123-32.

46. Grimsrud TK, Berge SR, Martinsen JI, Andersen A. Lung cancer incidence among Norwegian nickel-refinery workers 1953-2000. Journal of environmental monitoring : JEM. 2003;5(2):190-7.

47. Beveridge R, Pintos J, Parent ME, Asselin J, Siemiatycki J. Lung cancer risk associated with occupational exposure to nickel, chromium VI, and cadmium in two population-based case-control studies in Montreal. American journal of industrial medicine. 2010;53(5):476-85.

48. Sorahan T, Williams SP. Mortality of workers at a nickel carbonyl refinery, 1958-2000. Occupational and environmental medicine. 2005;62(2):80-5.

49. Luippold RS, Mundt KA, Austin RP, Liebig E, Panko J, Crump C, et al. Lung cancer mortality among chromate production workers. Occupational and environmental medicine. 2003;60(6):451-7.

50. Schubauer-Berigan MK, Deddens JA, Couch JR, Petersen MR. Risk of lung cancer associated with quantitative beryllium exposure metrics within an occupational cohort (English). Occupational and environmental medicine (London). 2011;68(5):354-60.

51. Schubauer-Berigan MK, Couch JR, Petersen MR, Carreon T, Yan JIN, Deddens JA. Cohort mortality study of workers at seven beryllium processing plants: update and associations with cumulative and maximum exposure (English). Occupational and environmental medicine (London). 2011;68(5):345-53.

52. Gowers DS, DeFonso LR, Schaffer P, Karli A, Monroe CB, Bernabeu L, et al. Incidence of respiratory cancer among workers exposed to chloromethyl-ethers. American journal of epidemiology. 1993;137(1):31-42.

53. Weiss W, Nash D. An epidemic of lung cancer due to chloromethyl ethers. 30 years of observation. Journal of occupational and environmental medicine / American College of Occupational and Environmental Medicine. 1997;39(10):1003-9.

54. Moulin JJ, Wild P, Romazini S, Lasfargues G, Peltier A, Bozec C, et al. Lung cancer risk in hard-metal workers. American journal of epidemiology. 1998;148(3):241-8.
